# Supplementary material for: Cancer/testis antigens FBXO39 and CEP55 expression correlates with survival in GBM patients
Source: PLoS One. 2025 Jun 12;20(6):e0326054. doi: 10.1371/journal.pone.0326054 (PMC12161592; doi:10.1371/journal.pone.0326054)
Supplement: Attachment — Submitted filename: Reviewers response.doc [file pone.0326054.s007.doc]

14 March 2024

**Dear Editorial Committee,**

*MS: No.*  PONE-D-24-09413

Cancer/testis antigens FBXO39 and CEP55 expression correlates with survival in GBM patients

Thank you for your e-mail. Please find the following point-by-point responses as requested:

**Comments to the Author**

1) Before we can continue with your submission, please upload a copy of the original ethics approval letter, as required by question 1 of the human subject research checklist. If the original letter is not in English, please also provide an English translated version in the supporting file. Please note that if this documentation is not included when your manuscript is resubmitted, it may be rejected.

Done

2) Thank you for submitting your work to PLOS ONE. Before we can proceed, please report in the Methods section the dates when data were accessed for research purposes. Please note that if this information is not included when your manuscript is resubmitted, it may be rejected.

The Methods Section was revised as suggested.

**Patients and tissue samples in our cohort**

This is a prospective study. From February 2020 to January 2023, 45 adults were operated for primary GBM at our Institute. Histologic classification of brain tumor samples using the current WHO CNS5 classification identified 29 glioblastomas, which were included in the study. Two normal brain tissue samples were used as controls in molecular analyses. Tumor and normal tissue samples were immediately frozen and stored at −80°C until use.

**Ethics statement.** All patients provided written consent to use their specimens for research purposes, none of them was identifiable. The study was approved by the Ethics Committee of the Shahid Beheshti University of Medical Sciences (Code: IR.SBMU.REC.1398.023, Tehran, Iran). The ethical principles of the Declaration of Helsinki were respected by researchers in all stages of the research.

3) Please complete all items on the Human Participants Research Checklist that are relevant for your submission, by following this link: <http://journals.plos.org/plosone/s/file?id=dc11/PLOSOne_Human_Subjects_Research_Checklist.docx>

Done.

I hope you find the corrections satisfactory.

Wish you all the best.

Yours sincerely

Azimi, P.
