# Supplementary material for: Cancer/testis antigens FBXO39 and CEP55 expression correlates with survival in GBM patients
Source: PLoS One. 2025 Jun 12;20(6):e0326054. doi: 10.1371/journal.pone.0326054 (PMC12161592; doi:10.1371/journal.pone.0326054)
Supplement: Attachment — Submitted filename: Reviewers_response_auresp_1.doc [file pone.0326054.s008.doc]

27 May 2024

**Dear Editorial Committee,**

*MS: No.*  PONE-D-24-09413

Cancer/testis antigens FBXO39 and CEP55 expression correlates with survival in GBM patients

Thank you for your e-mail. Please find the following point-by-point responses as requested:

**Comments to the Author**

**Reviewer #1:**

This article focuses on glioblastoma multiform, a type of tumor with poor prognosis, and seeks potential diagnostic and therapeutic biomarkers related to prognosis, which has certain clinical significance. The language of the article is clear, and the results are reliably substantiated.

Thank you.

However, there are still some shortcomings in the article:

1.The article only conducts survival-related analysis on genes that are highly expressed in tumor tissues. May it be possible to further search for other meaningful genes among significantly down-regulated genes.

We thank the referee for this very interesting suggestion.

This was revised as suggested.

a. The following sentence was added to the Results:

“The intersection between 279 CTA and the down-regulated genes of both TCGA and CGGA databases showed that 8 CTAs are decreased in GBM compared to healthy people, including SPAG6, CABYR, GPAT2, ELOVL4, TMEFF1, TMEFF2, MAGEC3, and SYCE1 (Figure 1b). “ and “ However, no significant effect was indicated for the remaining down-regulated CTAs regarding OS.”

b. The following sentence was added to the Discussion:

“Moreover, indeed, by nature, the CTA-encoding genes should be found only within the genes that are activated in cancer, since by definition, they are only expressed in the testis and not in normal somatic tissues. However, the brain is a particular tissue concerning the CTA encoding gene expression, since many testis-specific genes are known to be also expressed in the brain. We have also considered the list of significantly down-regulated genes compared to normal brain samples and checked if any of them is present in the list of CTA-encoding genes. We then considered the association between their down-regulation and the patient's survival. However, no statistically significant effect was observed on down-regulated CTAs regarding OS, albeit, more studies are needed to confirm the findings.“

2.The article finds that the expression levels of FBXO39 and CEP55 are related to prognosis in the analysis of public data, but in their own cohort, the P-values for both are greater than 0.05. It may be beneficial to increase the number of cohort for further validation.

The following sentence was added to the Discussion: “In this regard, it may be necessary to increase the number of cohort participants in future validation studies.”

3. The article only validates the high expression of FBXO39 and CEP55 at the RNA level in tumor tissues, lacking protein-level verification. It is suggested to supplement with immunohistochemical staining of paraffin sections.

We thank the referee for these suggestions, however, although companies are commercializing these antibodies, we could not identify antibodies already used in the pathology laboratories and directly usable in immunohistochemistry. However, we have this in mind, and at the first opportunity, we would test anti-FBXO39 and CEP55 antibodies if a reliable source is identified.

**Reviewer #2:**

Biomarker discovery in neuro-oncology has tremendous significance towards application in future therapies. Azimi et al. propose the utilization of cancer testis antigens as prospective biomarker candidates. Initially, the authors screen available RNA sequencing datasets from the United States and China. Specifically, the authors assess differentially expressed genes in glioblastoma (GBM) compared to normal brain. The authors then identified cancer testis genes that were specifically upregulated in the GBM cohorts and demonstrated correlation to patient survival. Following this discovery, Azimi et al. employed a prospective study of GBM patients, from their own institute, to validate a hypothesis that the expression of two testis antigens (FBXO39 and CEP55) correlates with patient survival. The manuscript is composed of two tables highlighting relevant clinical information and five figures. The main manuscript is supported by four spreadsheets featuring raw count data and statistics. The authors conclude that FBXO39 and CEP55 deserve consideration as biomarkers for GBM. While the prospective study is a unique dataset with substantial interest to the scientific community, the results underlying the authors' conclusion are exclusively correlative and lack necessary scientific rigor. Thus, the results, as currently written, do not merit the stated conclusion. Additionally, the written manuscript has components that may benefit from language editing. For these reasons, my recommendation would be to reject this manuscript.

The following are suggested minor revisions for the manuscript:

1. In paragraph 1 of the introduction, a comparison of patient characteristics and demographics from around the world would be beneficial. An example of one of these repositories is the US CBTRUS.

The following sentence was added to the Introduction: “Glioblastomas accounted for 60.2% of all gliomas, 14.2 % of all CNS tumors, and 50.9% of all malignant CNS tumors. Incidence of GBM increased slightly statistically significantly from 2000-2004 and 2004-2019. With an incidence rate of 3.27 per 100,000 persons in the United States, and more common in males, it is uncommon in children and increases with age [2]. The diverse factors such as incidence rates, tumor location, age of diagnosis, race, and the role of molecular genetics in the diagnosis of GBM, etc., are reported in various regions of the world. However, making comparisons of some data e.g., GBM incidence was complex due to significant differences in the methodology of epidemiological reports [3] “

2. Ostrom QT, Price M, Neff C, Cioffi G, Waite KA, Kruchko C, et al. CBTRUS Statistical Report: Primary Brain and Other Central Nervous System Tumors Diagnosed in the United States in 2016-2020. Neuro Oncol. 2023 Oct 4;25(12 Suppl 2):iv1-iv99.

3. Grochans S, Cybulska AM, Simińska D, Korbecki J, Kojder K, Chlubek D, Baranowska-Bosiacka I. Epidemiology of Glioblastoma Multiforme-Literature Review. Cancers (Basel). 2022;14(10):2412.

2. In the methods sections, please provide details on how normal brain tissue data was retrieved from TCGA and CGGA, including the number of normal specimens

This was revised as suggested in the Method: “ The RNA-seq and relevant clinical data of GBM patients, and normal controls were acquired from The Cancer Genome Atlas (TCGA, <https://www.cancer.gov/>) and the Chinese Glioma Genome Atlas (CGGA; [http://www.cgga.org](http://www.cgga.org/).cn/) database (CGGA.mRNAseq_693) [10]. A total of 418 GBM and 25 normal brain samples, including 169 and 5 from TCGA and 249 and 20 from the CGGA were included in the present study.“

3. In the statistical analysis subsection of methods, please describe, in detail, the stratification of high and low expression samples used in the PCR and subsequent Kaplan Meier analysis.

The following sentence was added to Methods:

“For expression and survival analysis, we employed the median gene value to categorize tumor samples into high and low-expression groups. Subsequently, the expression data were merged with clinical survival data using sample barcodes. GraphPad Prism was employed to model these two groups' survival time and status. Log-rank test was utilized for the comparison of Survival Curves. “

4. Related to manuscript editing, in the "gene expression and overall survival analysis in our cohort" subsection, the language is misleading. In earlier sections, correlation was interpreted with a poor survival phenotype. However, in this section, the correlation refers to prolonged survival. This interpretation is valid but misleading.

This was revised as suggested.

Additionally, the discussion section refers to the lack of correlation for some targets without presenting the necessary figure.

The following sentence was added to the Results:

“As shown in Figure 4, genes (CTCFL, PLAC1, LEMD1, PRAME, CEP55, DKKL1, PBK, ATAD2, and TTK) from the GEPIA2 dataset, and genes (PLAC1, FBXO39, LEMD1, DKKL1, PBK, ATAD2, and TTK) of primary and recurrent GBM from the mRNA_seq693 of the CCGA cohort were not associated significantly with overall survival in GBM patients. For gene (POTEGE), and genes (CTCFL, PRAME, and POTEG), Kaplan-Meier was not analyzed due to the small sample size in the GEPIA2 and CGGA datasets, respectively. “

Figure 4. (a–p) Kaplan-Meier analysis of overall survival for GBM patients in the GEPIA2 using the TCGA cohort (a. CTCFL, b. PLAC1, c. LEMD1, d. PRAME, e. CEP55, f. DKKL1, g. PBK, h. ATAD2, and i. TTK), and primary GBM from the mRNA_seq693 of the CCGA (j. PLAC1, k. FBXO39, l. LEMD1, m. DKKL1, n. PBK, o. ATAD2, and p. TTK) based on low- and high-expression of genes. The red line represents samples with high expression of the genes, and the blue line represents the samples with low expression of genes. Among 11 genes, p < 0.05 was considered to be statistically different.

5. For figures 2A/B (and 4 A/B), it may be beneficial to provide the actual p-value.

This was revised as suggested.

The following are suggested major revisions for the manuscript:

1. Even though it was mentioned in the text that the survival trend was insignificant, the manuscript would be improved by presenting Kaplan Meier survival data for a larger panel of testis antigens. Presenting this negative data for both US and Chinese cohorts may be enlightening towards a personalized medicine approach for other patient cohorts.

The following sentence was added to the Results:

“As shown in Figure 4, genes (CTCFL, PLAC1, LEMD1, PRAME, CEP55, DKKL1, PBK, ATAD2, and TTK) from the GEPIA2 dataset, and genes (PLAC1, FBXO39, LEMD1, DKKL1, PBK, ATAD2, and TTK) of primary and recurrent GBM from the mRNA_seq693 of the CCGA cohort were not associated significantly with overall survival in GBM patients. For gene (POTEGE), and genes (CTCFL, PRAME, and POTEG), Kaplan-Meier was not analyzed due to the small sample size in the GEPIA2 and CGGA datasets, respectively. “

Figure 4. (a–p) Kaplan-Meier analysis of overall survival for GBM patients in the GEPIA2 using the TCGA cohort (a. CTCFL, b. PLAC1, c. LEMD1, d. PRAME, e. CEP55, f. DKKL1, g. PBK, h. ATAD2, and i. TTK), and primary GBM from the mRNA_seq693 of the CCGA (j. PLAC1, k. FBXO39, l. LEMD1, m. DKKL1, n. PBK, o. ATAD2, and p. TTK) based on low- and high-expression of genes. The red line represents samples with high expression of the genes, and the blue line represents the samples with low expression of genes. Among 11 genes, p < 0.05 was considered to be statistically different.

2. For the interpretation of survival data, multivariate analysis is required. First, the CGGA dataset includes primary and recurrent GBM cases. Second, there is an age disparity that may confound the results. Logistic regression or other modeling should be employed.

The following sentence was added to the Methods:

“Cox regression analysis of factors contributing to overall survival of GBM patients

Overall survival was defined as the time from the start of treatment to death or last follow-up based on public databases. This overall survival time was the outcome of interest. The prognostic prediction ability of 11 gene expression, age, gender, and primary/recurrence status of GBM patients was examined using multivariate Cox regression based on samples from the TCGA and CGGA databases, separately. Hazard ratios (HRs) and 95% confidence intervals (CIs) were calculated accordingly. Then, factors with significant prognostic values were assessed. “ and “The multivariate Cox regression analyses were employed to recognize the independent clinical prognostic factors using the Cox regression in SPSS. “

The following sentence was added to the Results:

“The multivariate Cox regression analysis was performed to obtain the independent prognostic feature factors. As shown in Table 3-4 ( Supplementary files 5-6), factors were found to be independently correlated to OS prognosis in the TCGA and the CGGA datasets, respectively. However, only, the LEMD1 gene and primary/recurrence status were significant in the CGAA cohort.“

The following sentence was added to the Discussion:

“ One might inquire about the significant genes obtained from this study. Certainly, various computer modeling algorithms and prediction methods have been applied to predict outcomes in medical research. Selecting the best model is controversial and is dependent on the interest point of the research, and research in this field continues [29]. The most important variables will be changed by changing the algorithm structure [29]. Multivariable Cox regression analysis identified the LEMD1 gene and primary/recurrence status as independent prognostic factors for OS in the CGAA cohort. In contrast, statistical significance was not observed in variables in the TCGA dataset (Tables 3 and 4). On the other hand, as we all know, the TCGA and the CGGA databases are the world's largest and most comprehensive gene expression public databases in GBM patients. But, gene expression of CTCFL, POTEG, and PRAME was not reported in the CGGA dataset (Table 4; Supplementary 6). Moreover, the differences seen between the two databases can be due to the differences in genetics between the different populations. In this study, the significant genes were selected based on two different methods, therefore, the most important genes are different [29-30]. However, according to our study methods, CEP55 and FBXO39 genes were considered. “

29. Kimberley D. Brosofske, Robert E. Froese, Michael J. Falkowski, et al. A Review of Methods for Mapping and Prediction of Inventory Attributes for Operational Forest Management. Forest Science. 2014; 60(4): 733–756.

30. Fury W, Batliwalla F, Gregersen PK, Li W: Overlapping probabilities of top ranking gene lists, hypergeometric distribution, and stringency of gene selection criterion. Engineering in Medicine and Biology Society, 2006. EMBS'06. 28th Annual International Conference of the IEEE. 2006, IEEE, 5531-5534.

3. Both TCGA and CGGA datasets have low grade glioma information; the prospective study also has samples unused presumably based on a lower diagnosis grade. The prognostic value of the study would be improved with the inclusion of lower grade glioma data.

a. We thank the referee for these suggestions, however, the aim of this study in phase 1 is to identify and validate the key CTA genes, whose expression correlates with the survival of GBM patients. However, we have this in mind, and at the first opportunity, we will perform a phase 2 study about low-grade glioma.

b. The following sentence was added to the Discussion: “Secondly, the prognostic value of the study would be improved with the inclusion of lower-grade glioma data. However, low-grade glioma was not analyzed separately in the study due to the small sample size in our cohort.”

4. The utilization of this study to further assessment of these antigens as predictive markers in immunotherapy was mentioned in the introduction and discussion. Based on the scope of the project, if the authors do not want to assess patients treated with immunotherapy or assess immunotherapy targets pre-clinically, the innovation associated with predictive power in immunotherapy should be de-emphasized.

a. The following sentence was added to the Introduction: “ In addition, it is also possible that these specific antigens may be potential targets for immunotherapy in future clinical trials and pre-clinical research. “

b. The following sentence was added to the Discussion:

“Despite promising preclinical studies in several tumor types, however, the antitumor value of CTA‑based vaccines has been restricted in clinical trials [25]. Considering the difference of GBM from other tumors, there are still many limitations and difficulties in the application of CTAs in the treatment of GBM patients. This is mainly because of the unclear expression profile of most CTA in GBM [26]. However, targeting the FBXO39 and CEP55 genes may contribute to developing novel GBM-specific biomarkers in future clinical trials and pre-clinical research. “

26. Li F, Liu C, Nong W, Lin L, Ge Y, Luo B, et al. Identification of potential biomarkers in cancer-testis antigens for glioblastoma. Am J Transl Res. 2023;15(2):799-816.

c. The following sentence was added to the Discussion: “Overall, large preclinical and clinical studies are needed to assess targeting for FBXO39 and CEP55 in GBM patients. “

5. Similar to major revision #1, a larger panel of cancer-testis antigens should be tested using the same PCR system.

The following sentence was added to the Discussion: “Thirdly, it is recommended that genes ( CTCFL, PLAC1, LEMD1, PRAME, POTEG, DKKL1, PBK, ATAD2, and TTK) can be tested using the same PCR system with a larger sample size. “

The authors' current conclusion is misleading without further rigorous validation. However, the authors identified an interesting finding. If explored further, the current work has strong potential to inform the neuro-oncology community. Specifically, the current data shows FBXO39 correlates with patient survival in the United States and CEP55 correlates with patient survival in China. Intriguingly, both these antigens appears to correlate with survival in the Middle Eastern cohort. Based on a multitude of factors (age, ethnicity, TCGA transcriptional subclass), it is difficult to interpret this observation but further analysis can illuminate a rationale for this observation. Generating a cancer testis signature by patient stratification, either by age at diagnosis or demographic, has tremendous potential as a biomarker. Nevertheless, trends in neuro-oncology stipulate that brain tumors are a unique entity (dot: 10.1158/2159-8290.CD-23-1498), unrelated to other cancers and tissues. Considering this interpretation, cancer testis antigens may not be informative in the clinical diagnosis, or treatment, of GBM.

a. The following sentence “The expression of CTAs of FBXO39 and CEP55 was high in GBM patients. It can be concluded that these markers could be ideal targets for immunotherapy in such patients. FBXO39 and CEP55 can be considered important markers in determining the survival of GBM patients. “ was changed to “ The expression of CTAs of FBXO39 and CEP55 was high in GBM patients. FBXO39 and CEP55 can be considered important markers in determining the survival of GBM patients. However, further study is needed with a larger sample size to validate these findings and other cancer-testis antigens in correlation with survival in GBM patients. It is important to understand that GBMs differ both biologically and clinically from other tumors. Hence, survival outcomes assessment of GBM needs to be approached from different angles given its heterogeneity, genomic and epigenetic state, localization, race, tumor size, age of diagnosis, etc. “ in the Conclusion.

b. the following sentence was added to the Discussion:

“This study identified an interesting finding. If explored further, the current work has strong potential to inform the neuro-oncology community. Specifically, the current data shows FBXO39 correlates with patient survival in the United States and CEP55 correlates with patient survival in China. Intriguingly, both these antigens appear to correlate with survival in the Middle Eastern cohort. Based on a multitude of factors (age, ethnicity, TCGA transcriptional subclass), it is difficult to interpret this observation but further analysis can illuminate a rationale for this observation [3]. Generating a cancer-testis signature by patient stratification, either by age at diagnosis or demographic, has tremendous potential as a biomarker. Nevertheless, trends in neuro-oncology stipulate that brain tumors are a unique entity unrelated to other cancers and tissues [28]. Considering this interpretation, cancer-testis antigens may not be informative in the clinical diagnosis, or treatment, of GBM. “

I hope you find the corrections satisfactory.

Wish you all the best.

Yours sincerely

Azimi, P.
