# Supplementary material for: Cancer/testis antigens FBXO39 and CEP55 expression correlates with survival in GBM patients
Source: PLoS One. 2025 Jun 12;20(6):e0326054. doi: 10.1371/journal.pone.0326054 (PMC12161592; doi:10.1371/journal.pone.0326054)
Supplement: Attachment — Submitted filename: Reviewers_response_auresp_2.doc [file pone.0326054.s009.doc]

2 August 2024

**Dear Editorial Committee,**

*MS: No.*  PONE-D-24-09413R1

Cancer/testis antigens FBXO39 and CEP55 expression correlates with survival in GBM patients

Thank you for your e-mail. Please find the following point-by-point responses as requested:

**Comments to the Author**

1. If the authors have adequately addressed your comments raised in a previous round of review and you feel that this manuscript is now acceptable for publication, you may indicate that here to bypass the “Comments to the Author” section, enter your conflict of interest statement in the “Confidential to Editor” section, and submit your "Accept" recommendation.

**Reviewer #1**: All comments have been addressed

**Reviewer #3**: All comments have been addressed

Thank you.

2. Is the manuscript technically sound, and do the data support the conclusions?

The manuscript must describe a technically sound piece of scientific research with data that supports the conclusions. Experiments must have been conducted rigorously, with appropriate controls, replication, and sample sizes. The conclusions must be drawn appropriately based on the data presented.

**Reviewer #1**: Yes

**Reviewer #3**: Yes

3. Has the statistical analysis been performed appropriately and rigorously?

**Reviewer #1**: Yes

**Reviewer #3**: I Don't Know

4. Have the authors made all data underlying the findings in their manuscript fully available?

The [PLOS Data policy](http://www.plosone.org/static/policies.action#sharing) requires authors to make all data underlying the findings described in their manuscript fully available without restriction, with rare exception (please refer to the Data Availability Statement in the manuscript PDF file). The data should be provided as part of the manuscript or its supporting information, or deposited to a public repository. For example, in addition to summary statistics, the data points behind means, medians and variance measures should be available. If there are restrictions on publicly sharing data—e.g. participant privacy or use of data from a third party—those must be specified.

**Reviewer #1**: Yes

**Reviewer #3**: Yes

5. Is the manuscript presented in an intelligible fashion and written in standard English?

PLOS ONE does not copyedit accepted manuscripts, so the language in submitted articles must be clear, correct, and unambiguous. Any typographical or grammatical errors should be corrected at revision, so please note any specific errors here.

**Reviewer #1**: Yes

**Reviewer #3**: Yes

6. Review Comments to the Author

Please use the space provided to explain your answers to the questions above. You may also include additional comments for the author, including concerns about dual publication, research ethics, or publication ethics. (Please upload your review as an attachment if it exceeds 20,000 characters)

**Reviewer #1:** The author has proposed good improvement plans and corresponding explanations for the raised issues. No further concerns are related.

Thank you.

**Reviewer #3:** This study explores the potential of FBXO39 and CEP55 as prognostic biomarkers for GBM. Utilizing RNA-seq data from the TCGA and CGGA databases, the study identifies differentially expressed genes and specifically highlights up-regulated cancer/testis antigens (CTAs) that correlate with overall survival in GBM patients. The findings are validated with an independent cohort of 29 GBM patients using RT-qPCR. The study concludes that high expression levels of FBXO39 and CEP55 are significantly associated with poorer overall survival, suggesting these genes as potential targets for prognostic assessment and therapeutic intervention in GBM. Further research with larger sample sizes and protein-level validation is recommended to strengthen these findings.
Title:

The title is clear and accurately reflects the content of the manuscript.
Abstract:

The abstract provides a good summary of the study's background, methods, results, and conclusions.

The mention of both databases (TCGA and CGGA) and the independent cohort is good, providing a clear understanding of the study's scope.

Consider revising sentences for conciseness and clarity.

This was revised as suggested.

Introduction

The introduction provides a clear background on GBM and the significance of cancer/testis antigens (CTAs) as biomarkers.

The inclusion of statistics and references to WHO classification and incidence rates enhances the context.

The following sentence was added to the Introduction”

“The classification of brain tumors has undergone several changes over time due to various factors such as genetic variations. The World Health Organization (WHO) has played a key role in the effort [1].”

The following sentence was revised as suggested:

“With an incidence rate of 3.27 per 100,000 persons in the United States, and more common in males, it is uncommon in children and increases with age [2].”

The rationale for studying FBXO39 and CEP55 is well-explained, linking it to the potential for novel therapeutic targets.

Methods

The methods section is detailed and logically structured.

Descriptions of data acquisition, DEG screening, and identification of CTAs are clear.

The explanation of survival analysis and the use of independent cohorts are well-detailed.

The addition of the Cox regression analysis for overall survival is a good inclusion, addressing one of the reviewer’s concerns.

Consider breaking down long paragraphs into smaller sections for better readability.

This was revised as suggested.

The following sentence “ The prognostic prediction ability of 11 gene expression, age, gender, and primary/recurrence status of GBM patients was examined using multivariate Cox regression based on samples from the TCGA and CGGA databases, separately. “ was changed to “Multivariate Cox regression included 11 gene expressions, age, gender, and primary/recurrence status of GBM patients. Samples from the TCGA and CGGA databases, separately, were used. “

Results

The results are comprehensive and clearly presented.

Figures and tables support the textual data effectively.

Consider revising sentences for conciseness and clarity.

This was revised as suggested.

Discussion

The discussion contextualizes the findings within existing literature well.

The implications of FBXO39 and CEP55 as prognostic markers are clearly outlined.

Consider addressing the need for protein-level validation more directly, as it was a significant reviewer comment.

The following sentence was added to the Discussion:

“We could not identify antibodies already used in the pathology laboratories and directly usable in immunohistochemistry. However, we have this in mind, and at the first opportunity, we would test anti-FBXO39 and CEP55 antibodies if a reliable source is identified.”

Conclusion

The conclusion summarizes the key findings and their potential implications succinctly.

The call for further studies with larger sample sizes is appropriate and necessary.

Overall Assessment

Clarity: The manuscript is generally clear, but some sentences can be revised for better readability and conciseness. Breaking down longer sections in the Methods and Results can also improve clarity.

This was revised as suggested.

Conciseness: The manuscript is concise overall but can benefit from removing redundant phrases and combining shorter sentences where possible.

This was revised as suggested.

Figures and Tables: Effective use of visual aids supports the findings.

References: The references are comprehensive and relevant.

Based on the clarity and conciseness, as well as the significant revisions made in response to reviewers' comments, this manuscript is suitable for publication with minor revisions. Addressing the suggestions for improving readability and ensuring grammatical precision will further enhance the manuscript's quality.

This was revised as suggested.

I hope you find the corrections satisfactory.

Wish you all the best.

Yours sincerely

Azimi, P.
