# Supplementary material for: Cancer/testis antigens FBXO39 and CEP55 expression correlates with survival in GBM patients
Source: PLoS One. 2025 Jun 12;20(6):e0326054. doi: 10.1371/journal.pone.0326054 (PMC12161592; doi:10.1371/journal.pone.0326054)
Supplement: Attachment — Submitted filename: Reviewers_response_auresp_3.doc [file pone.0326054.s010.doc]

8 December 2024

**Dear Editorial Committee,**

*MS: No.* PONE-D-24-09413R2

Cancer/testis antigens FBXO39 and CEP55 expression correlates with survival in GBM patients

Thank you for your e-mail. Please find the following point-by-point responses as requested:

**Comments to the Author**

1. If the authors have adequately addressed your comments raised in a previous round of review and you feel that this manuscript is now acceptable for publication, you may indicate that here to bypass the “Comments to the Author” section, enter your conflict of interest statement in the “Confidential to Editor” section, and submit your "Accept" recommendation.

Reviewer #4: All comments have been addressed

Thank you.

2. Is the manuscript technically sound, and do the data support the conclusions?

The manuscript must describe a technically sound piece of scientific research with data that supports the conclusions. Experiments must have been conducted rigorously, with appropriate controls, replication, and sample sizes. The conclusions must be drawn appropriately based on the data presented.

**Reviewer #4:** Partly

3. Has the statistical analysis been performed appropriately and rigorously?

Reviewer #4: Yes

4. Have the authors made all data underlying the findings in their manuscript fully available?

The PLOS Data policy requires authors to make all data underlying the findings described in their manuscript fully available without restriction, with rare exception (please refer to the Data Availability Statement in the manuscript PDF file). The data should be provided as part of the manuscript or its supporting information, or deposited to a public repository. For example, in addition to summary statistics, the data points behind means, medians and variance measures should be available. If there are restrictions on publicly sharing data—e.g. participant privacy or use of data from a third party—those must be specified.

**Reviewer #4:** Yes

5. Is the manuscript presented in an intelligible fashion and written in standard English?

PLOS ONE does not copyedit accepted manuscripts, so the language in submitted articles must be clear, correct, and unambiguous. Any typographical or grammatical errors should be corrected at revision, so please note any specific errors here.

**Reviewer #4:** Yes

6. Review Comments to the Author

Please use the space provided to explain your answers to the questions above. You may also include additional comments for the author, including concerns about dual publication, research ethics, or publication ethics. (Please upload your review as an attachment if it exceeds 20,000 characters)

**Reviewer #4:** Thank you for inviting me to review the article. In this article, the authors explored cancer testis antigen genes FBXO39 and CEP55 and their correlation with survival in GBM patients, using TCGA and CGGA databases and multiple bioinformatic methods. I generally think that the article matches the journal's scope and quality. However, there are several questions that I would like authors to address before I can be fully convinced.

1. Figure 2a and Figure 2b showed that both FBXO39 and CEP55 were significantly increased in TCGA data. However, there is a huge gap in case numbers between normal and GBM patients, 5 of normal vs. 169 of GBM. Is this comparison trustable? Actually as I tried to analyze using TCGA-GTEx (there are 207 cases in GTEx group, representing normal brain tissue), there was no significance between TCGA and normal. Therefore, I would like the authors to explain why they chose to use TCGA data only instead of including GTEx data to make the result of NORMAL more representative.

We appreciate the reviewer's insightful comments regarding the disparity in sample sizes between normal (5 samples) and GBM (169 samples) patients in the TCGA dataset.

Our decision to utilize TCGA data exclusively for this analysis was based on several factors:

**Matched Samples:** The normal samples from TCGA are derived from adjacent tissues of cancer patients, which may provide relevant biological context compared to completely healthy tissue samples. However, we acknowledge that these samples may carry some tumor microenvironment signals, potentially influencing gene expression levels.

**Statistical Power:** While the number of normal samples is limited, TCGA's extensive dataset for GBM provides a robust basis for statistical analysis, allowing us to identify significant differences in gene expression that may be clinically relevant.

We recognize that incorporating GTEx data could enhance the representativeness of normal brain tissue comparisons.

To address the reviewer's suggestion, we conducted additional analyses that include GTEx data.

According to the dear reviewer's opinion, we used the set of normal GTEx data and normal TCGA samples. The results of our analyzes using gepia2 (http://gepia2.cancer-pku.cn/#analysis) showed that the CEP55 expression in GBM samples is increased compared to normal tissue but the FBXO39 expression in GBM samples was not significantly different compared to normal tissue. (You can see the results below).

This discrepancy may arise from differences in sample processing and batch effects between the two datasets, as highlighted in existing literature 1, 2, 3.

1. <https://www.nature.com/articles/sdata201861>

(Unifying cancer and normal RNA sequencing data from different sources)

2. <https://ucsc-xena.gitbook.io/project/how-do-i/tumor-vs-normal>

(How do I compare tumor vs normal expression?)

3. <https://www.biostars.org/p/9502850/>

(Combining GTEx and TCGA data)

We appreciate the reviewer’s recommendation to include GTEx data for a more comprehensive analysis. This will not only strengthen our results but also provide a clearer understanding of gene expression dynamics in GBM relative to healthy brain tissue.

We hope this response clarifies our rationale for using TCGA data and outlines our commitment to enhancing the robustness of our findings through further analysis. Thank you for your valuable feedback.

FBXO39:

CEP55:

2. As I checked on GDC data portal, there are 617 cases in TCGA-GBM project. The authors used 169 cases from TCGA. Can authors explain their selection criteria of TCGA cases? How did they avoid selection bias?

We sincerely thank the reviewer for his thorough observations. While it is true that the GDC data portal lists a total of 617 cases, this figure encompasses all GBM samples with various analyses performed on these samples, including Copy Number Variation, miRNA-Seq, DNA Methylation, and RNA-Seq. we specifically focused on GBM RNA-seq data, along with 5 normal samples obtained from adjacent tissues of cancer patients. We have included the following command to facilitate the download of data related to the TCGA-GBM project.

We used the TCGAbiolinks R package to download GBM RNAseq data.

Query command = GDCquery (project = "TCGA-GBM", data.category = "Transcriptome Profiling" data.type = "Gene Expression Quantification",

workflow.type = "STAR - Counts",

legacy = F, experimental.strategy = "RNA-Seq")

7. PLOS authors have the option to publish the peer review history of their article (what does this mean?). If published, this will include your full peer review and any attached files.

If you choose “no”, your identity will remain anonymous but your review may still be made public.

Do you want your identity to be public for this peer review? For information about this choice, including consent withdrawal, please see our Privacy Policy.

**Reviewer #4:** No

I hope you find the corrections satisfactory.

Wish you all the best.

Yours sincerely

Azimi, P.
